# Design of a Microflyer Driven by a Microsized Charge Combined with an Initiation Criterion

**DOI:** 10.3390/mi14020312

**Published:** 2023-01-26

**Authors:** Xiang He, Lixin Yang, Haiping Dong, Zhixing Lv, Nan Yan

**Affiliations:** 1 State Key Laboratory of Explosion Science and Technology, School of Mechatronical Engineering, Beijing Institute of Technology, Beijing 100081, China; 2 Beijing Institute of Space Mechanics & Electricity, Beijing 100094, China; 3 China Safety Technology Research Academy of Ordnance Industry, Beijing 100053, China

**Keywords:** microdetonation sequence, lead azide, flyer, initiation criterion

## Abstract

In order to study the performance of ultra-fine 2,2′, 4,4′, 6,6′–hexanitrostilbene (HNS-IV) explosives initiated by a microflyer driven by microsized lead azide (Pb(N_3_)_2_), a corresponding simulation model was established in Autodyn software, and the accuracy of the simulation model was verified with a photonic Doppler velocimeter (PDV). Various influencing factors were studied in combination with the power flux–action time (*Π*-*τ*) initiation criterion. The results showed that the exponential growth rate of the flyer velocity decreased with an increase in the diameter and height of the lead azide and that the influence of the charge diameter was more obvious than that of the charge height. The flyer velocity increased linearly with the density of the lead azide. The velocity of the flyer also increased linearly with an increase in the shock wave impedance of the restraint materials, and the velocities of the flyer that corresponded to silicon and organic glass were lower than those of the metal materials. The flyer’s velocity and power flux increased with a decrease in the flyer’s density; when considering the flyer’s velocity, power flux, and actual shear effect, titanium was the best material for the flyer. As the thickness of the flyer was decreased, the velocity and power flux of the flyer increased; under the premise of satisfying the forming effect, the thinner flyer was selected. When used as the material for the acceleration chamber, silicon showed a lower flyer velocity and power flux than sapphire, nickel, stainless steel, and other materials. With the increase in the acceleration chamber aperture, the exponentially declining trend in the flyer’s velocity increased; when the aperture of the accelerating chamber was consistent with the diameter of the primary explosive, the power flux was the largest. Finally, the ability of the microflyer to initiate the HNS-IV was verified by a steel dent test.

## 1. Introduction

With the emergence of small-caliber intelligent ammunition and unmanned and small-load combat platforms, fuses have more application requirements with respect to the miniaturization of detonation sequences [1]. A reduction in volume of a detonation sequence provides more space for the ammunition warhead and circuit design. A flyer-type detonation sequence uses the detonation products of the primary microexplosive to drive the microflyer to detonate the booster explosive. The flyer-type detonation sequence is smaller than the volume and charge amount of the detonator. Moreover, the flyer can maintain a higher velocity within several millimeters, which is significantly lower than the energy-attenuation rate of the shock wave; at the same time, the safe and arm (S&A) devices no longer need a special-shaped booster, so the structure of the detonation sequence is simpler.

Gerald proposed three design schemes of the detonation sequence of flyers driven by in situ explosives to initiate booster explosives: a linear size of the detonation sequence of less than 1 mm and a charge of less than 10 mg, deposition of a plastic flyer (p-xylene, polyimide, etc.) on the initiating explosive layer, and etching through holes on the back of the base as acceleration chamber. Cyclotrimethylene trinitramine (RDX), HNS, pentaerythritol tetranitrate (PETN), cyclotetramethylene tetranitramine (HMX)/Viton A–95/5 (PBXN-5), triaminotrinitrobenzene (TATB)/RDX/Viton-60/35/5 (PBXN-7), or PETN/silicone resin-80/20 (PBXN-301) were detonated after the flyer was accelerated using in situ explosives [2]. The U.S. Army designed a micro detonation sequence of a titanium flyer driven by a hexanitrohexaazaisowurtzitane (CL-20)-based ink explosive (EDF-11) to detonate the booster explosives [3,4,5]. The micro detonation sequence designed by the U.S. Navy used pressed or in situ silver azide (Ag(N_3_)_2_, φ 2 mm × 0.5 mm, and 6 mg) or in situ copper azide (Cu(N_3_)_2_) as primary explosives; the stainless steel flyer (0.3 mm~1 mm thick) was accelerated to detonate EDF-11 explosives, and the EDF-11 was laterally exported to generate a shock wave or drive the flyer to detonate CL-20/proprietary binders-97.75/2.25 (RSI-007)/PBXN-5 [6,7,8]. A micro detonation sequence for a 25 mm~40 mm grenade was proposed by the American Kaman Company; the rotor carries Pb(N_3_)_2_ (a slurry charge) to the release position, which is aligned with the lead styphnate (LTNR) of the igniter; the Pb(N_3_)_2_ shears the nickel-cobalt alloy to form a flyer; and the flyer accelerates in the base of booster and detonates the booster HNS-IV [9]. Wang’s research showed that lead azide with a size of φ 1.6 mm × 2 mm (with a weight of approximately 14 mg) could drive a stainless steel flyer that was 30 μm thick to successfully initiate HNS-IV [10]. Xie proposed a micro detonation sequence of a titanium flyer driven by Cu(N_3_)_2_; the CL-20 based booster explosive could be reliably detonated when the Cu(N_3_)_2_ weighed about 7.5 mg [11,12]. Tan used a simulation to analyze the speeds of flyers composed of different materials and thicknesses using different densities and sizes of tetraammine-cis-bis (5-nitro-2*H*-tetrazolato-*N*^2^) cobalt (III) perchlorate (BNCP) [13]. Zeng proposed a micro detonation sequence of HNS-IV initiated by a titanium flyer that was driven by microsized copper azide and found that the diameter and thickness of the charge and the length of the accelerating chamber significantly affected the velocity of the flyer and that the material of flyer was related to the shape of the flyer [14,15,16].

The azide-type primary explosive has a short detonation distance and certain power, and it is often used as the primary charge in a detonator. The output performance of microsized lead azide was proved [17,18], and the pressing characteristics of microsized lead azide was studied [19]. Copper azide and silver azide are relatively sensitive, while the sensitivity of lead azide is relatively low, making it the most widely used primary explosive at present. There is little research on the mechanism of microflyers driven by microsized lead azide.

The velocity of a flyer is an important parameter when measuring the detonation ability, but whether the booster explosive can be detonated is also related to the type of booster explosive; the material, shape, and size of the flyer; and other factors. The impact initiation criterion, which is related to the properties of the flyer and explosive, the velocity of the flyer, and the structural parameters, is a reliable tool that can be used to judge whether the flyer successfully detonated the booster explosive. The pressure peak, duration, and particle velocity of the incident shock wave are used to analyze the initiation of the booster explosive.

**Figure 1:**
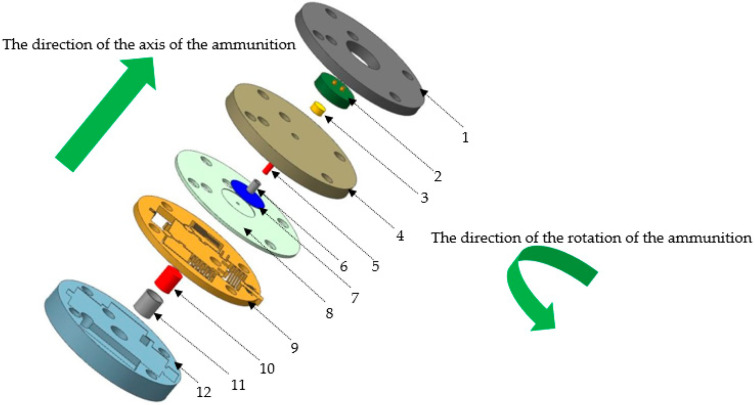
Schematic diagram of a microelectromechanical system (MEMS) detonation device: 1. sleeve of ignition unit; 2. semiconductor bridge unit; 3. ignition composition; 4. sleeve of primary explosive; 5. primary explosive; 6. shell of primary explosive; 7. titanium sheet; 8. upper cover plate of the S&A device; 9. structural layer of the S&A device; 10. booster explosive; 11. shell of booster explosive; 12. base.

Figure 1 shows a structural diagram of a MEMS detonation system. Such systems are usually composed of a microdetonation sequence and a micro S&A device. The ignition layer is composed of a semiconductor bridge and a lead styphnate, which can ignite lead azide, and the charge of the LTNR weighs a milligram. The primary explosive is lead azide, which is pressed with several milligrams of charge and is a millimeter in size. When the S&A device is armed, the detonation product of the primary explosive shears the titanium flyer, which accelerates in the shear hole of the upper cover plate of the micro S&A device and the detonation hole of the micro S&A device, and then detonates the HNS-IV booster explosive. In this study, we examined the relationship between the important influencing factors of a microflyer driven by microsized lead azide and the initiation ability based on the initiation criterion.

## 2. Materials and Methods

### 2.1. Materials

In this study, a microscale semiconductor bridge (SCB) was used for ignition. The material of the SCB was monocrystalline silicon with a size of 20 × 50 × 4 μm at 60°. The doping element was phosphorus, the doping concentration was 4 × 10^19^·cm^−3^, and the resistance value was about 4 Ω~5 Ω. It was fabricated using a deep reactive ion etching (DRIE) process in the micro/nano laboratory of Peking University.

The proportion of ignition agent was: 1 g weight of LTNR; 6% concentration of polyvinyl acetate, and 0.5 mL~1 mL. The average particle size after drying was about 70 μm, and the particles had a hexagonal prism shape. The production process of LTNR was as follows: sodium styphnate was prepared via the reaction of styphnic acid and sodium bicarbonate, and LTNR was formed via the reaction of sodium styphnate and lead(II) nitrate. The styphnic acid (chemically pure) was acquired from Xi’an Qinghua Co., Ltd. (Xi’an, China); lead(II) nitrate, an analytical reagent (AR), was acquired from Xilong Chemical Co., Ltd. (Shantou, China); the sodium bicarbonate (AR) was acquired from Tianjin Dengfeng Chemical Reagent Factory (Tianjin, China); and the polyvinyl acetate (AR) was acquired from Hubei Guangao Biotechnology Co., Ltd. (Tianmen, China). The ignition agent was applied to the SCB, aired for 20 min, and then applied again; after 60 min of airing, it was dried at 50 °C~60 °C for 4 h.

The primary explosive was carboxymethyl cellulose-lead azide (CMC-Pb(N_3_)_2_) with an average particle size of 5 μm and a columnar shape. Lead(II) acetate solution with a concentration of 14% and sodium azide solution with a concentration of 5.5% were added to a solution composed of 0.1% sodium carboxymethyl cellulose solution and 0.1% sodium tartrate solution. After the reaction, the solution was filtered, washed, and dried. The lead(II) acetate (AR) was acquired from Sinopharm Chemical Reagent Co., Ltd. (Shanghai, China); the sodium azide (AR) was acquired from Shanghai Qiyuan Biotechnology Co., Ltd. (Shanghai, China); the sodium carboxymethyl cellulose (AR) was acquired from Jiangsu Taize Biotechnology Co., Ltd. (Suzhou, China); and the sodium tartrate (AR) was acquired from Tianjin Jinhui Taiya Chemical Reagent Co., Ltd. (Tianjin, China). The lead azide was weighed multiple times using an electronic balance (with an accuracy of 0.1 mg), and multiple pressings were performed by using a small pressing mold (for submillimeter pressing) and a precision servo press machine (the pressure, speed, and time could be set in the program with an accuracy of ± 1 N). Finally, the lead azide was pressed into a micro tube shell.

The average particle size of the booster HNS-IV was 0.8 μm in a long rod shape. The HNS was used as the raw material and dimethylformamide was used as the solvent (1 g of HNS was added to 6 mL of dimethylformamide solvent). The HNS was dissolved by heating and then purified via recrystallization at a low temperature; and then the HNS-IV was obtained via mechanical grinding. The HNS (technically pure) was acquired from Xi’an Qinghua Co., Ltd. (Xi’an, China); and the dimethylformamide (AR) was acquired from Shaanxi Xintong Chemical Co., Ltd. (Xi’an, China). The HNS-IV was pressed into the tube shell at one time using a hand press machine.

### 2.2. Criteria of Flyer Impact Initiation

At present, the common initiation criteria are the *p*-*τ* criterion, the James criterion, and the *Π*-*τ* criterion. The theories and fitting methods of these three initiation criteria are introduced below.

#### 2.2.1. Theory of the *p-τ* Initiation Criterion

On the basis of energy conservation, Walker and Wasely proposed the concept of the critical initiation energy of explosives. After the flyer strikes the explosive, the explosive is detonated when the energy *E* loaded on the unit area of the explosive is greater than the critical initiation threshold energy density *E*_c_ [20] (*E*_c_ is a constant related to the properties of the explosive):

*E* = *puτ* (1)

where *E* represents the energy density in MJ·m^−2^, *p* represents the pressure in Gpa, *u* represents the particle velocity in km·s^−1^, and *τ* represents the action time of the incident shock wave in μs. Table A1 in Appendix A lists the main symbols in this study and the corresponding explanations and units. The relation between *p*, *D*, *ρ*, and *u* is:

*p* = *ρDu* (2)

where *D* represents the shock wave velocity in km·s^−1^, and *ρ* represents the density in g·cm^−3^. Equation (2) can be substituted into Equation (1) to obtain:

(3) $$E=\frac{p^{2}\tau }{\rho D}$$

The critical condition for initiation of explosives is:

(4) $$E_{c}=\frac{p^{2}\tau }{\rho D}$$

When the shock wave pressure produced by the impact of the flyer on the explosive changes slightly, the shock wave velocity *D* inside the explosive is constant, and the shock wave impedance *ρD* of the explosive can be considered as a constant [21]. The initiation criterion is:

*p*^2^*τ* = *C*_ini_ (5)

The initiation criterion *C*_ini_ is a constant related to the properties of explosives. For high-energy heterogeneous explosives, *D* is used in the power function of *p*, and the initiation criterion is [21]:

*p^n^τ* = *C*_ini_ (6)

In Formula (6), *n* is a constant related to the properties of explosives. When *p*^*n*^*τ* > *C*_ini_, it means that the explosive is detonated; on the contrary, it means that the explosive cannot be detonated. The shock initiation of explosive is related to the pressure *p* and the shock wave action time *τ* [22].

#### 2.2.2. Theory of the James Initiation Criterion

On the basis of the *p*-*τ* initiation criterion, James proposed an initiation criterion that is applicable to larger impact pressures, wider types of explosives, and more shapes of the flyer. The concept of the particle specific kinetic energy *Σ* is proposed as *Σ* = *u*^2^/2, MJ·kg^−1^.

The James initiation criterion can be expressed as [23]:

(7) $$\frac{\Sigma_{c}}{\Sigma }+\frac{E_{c}}{E}=1$$

In Formula (7), *Σ*_c_ is a constant related to the properties of explosives. The energy density *E*_c_ and particle specific kinetic energy *Σ*_c_ jointly determine whether the explosive detonates. When *Σ*_c_/Σ + *E*_c_/*E* > 1, it means that the explosive is detonated; otherwise, it means that the explosive cannot be detonated.

#### 2.2.3. Theory of the *Π-τ* Initiation Criterion

On the basis of James’s research, Welle and Kim proposed the concept of power flux *Π* where *Π* = *pu* in GW·cm^−2^. The power flux *Π* is used instead of the particle specific kinetic energy *Σ*. The power flux *Π* is the derivative of the energy density *E* for the action time *τ*, which is not only related to the impact strength but also represents the energy rate transmitted to the explosive per unit area.

The *Π*-*τ* criterion, which is defined by the energy density *E*, power flux *Π,* and action time *τ*, can be expressed as [24,25]:

(8) $$\Pi =\Pi_{c}+(\frac{E_{c}/\Pi_{c}}{\tau })$$

In Formula (8), *Π*_c_ is a constant related to the properties of explosives. When *Π* > *Π*_c_ + (*E*_c_/*τΠ*_c_), the explosive is detonated; otherwise, the explosive cannot be detonated.

#### 2.2.4. Parameter-Fitting Process of the Three Initiation Criteria for HNS-IV

The calculation method of the shock wave parameters and the fitting process of the initiation criteria of the HNS-IV are introduced below. The formula to calculate the pressure p and particle velocity u of the incident shock wave is [21]:

*p*_e_ = *p*_f_ = *ρ*_e0_(*A*_e_ + *S*_e_*u*_e_)*u*_e_ = *p*_f_[*A*_f_ + *S*_f_(*v*_f_ − *u*_e_)](*v*_f_ − *u*_e_) (9)

*u*_e_ = *v*_f_ − *u*_f_ (10)

where subscripts e and f represent explosives and flyers, respectively; *A* represents the intercept of the Hugoniot line in km·s^−1^; *S* represents the slope of the Hugoniot line (dimensionless); and *v*_f_ is the flyer velocity in km·s^−1^.

The calculation formulas of the action time of the explosives for flyers with different shapes are different [23]. The action time of the shock wave of the flat-nosed rod-shaped flyer is:

(11) $$\tau =\frac{d_{f}}{6C_{e}}$$

The action time of the shock wave of the sphere/round-nosed rod-shaped flyer is:

(12) $$\tau =\frac{d_{f}}{18C_{e}}$$

The action time of the shock wave of the plate-shaped flyer is:

(13) $$\tau =\frac{2\delta_{f}}{D_{f}}$$

where *d*_f_ represents the diameter of the flyer in μm, *C*_e_ represents the sound velocity inside the explosive in km·s^−1^, *δ*_f_ represents flyer thickness in μm, and *D*_f_ is the shock wave velocity in the flyer in km·s^−1^.

The first three columns and the last three columns of Table 1 provide the respective experimental data of the critical velocities for initiating HNS-IV using polyimide and aluminum flyers with different sizes. According to the velocity and size of the flyer, the Hugoniot parameters of the flyer and HNS-IV in Table 2, the pressure *p*, particle velocity *u*, and action time *τ* of the incident shock wave in the HNS-IV are calculated by using the Formula (9)~(12), and then the energy density *E*, specific kinetic energy *Σ*, and power flux *Π* are obtained.

**Figure 2:**
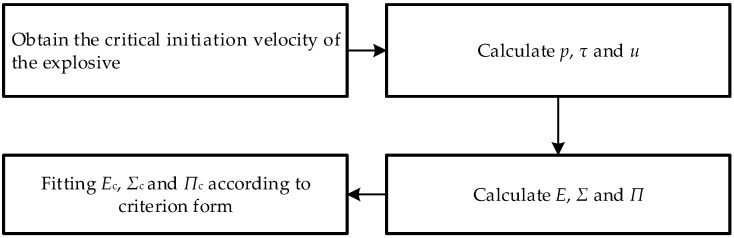
The fitting process of the initiation criterion.

The parameter-fitting process of the initiation criterion is shown in Figure 2.

The Hugoniot parameters of the booster explosive and flyers are shown in Table 2.

### 2.3. Simulation of The Microflyer Driven by Microsized Lead Azide

#### 2.3.1. Design of Simulation

In order to provide data support for the parameter design of the microdetonation sequence and to study the laws of a flyer driven by a microcharge, a simulation model of a microflyer driven by microsized lead azide was established, and the velocity of the flyer corresponding to the microdetonation sequence with different structural parameters was obtained. Based on Section 2.2, parameters such as the pressure *p*, particle velocity *u*, and action time *τ* could be calculated. Furthermore, the relationship between the structural parameters of the microdetonation sequence and the initiation ability of the flyer was analyzed in combination with the shock initiation criterion.

Studying the relationship between the key influencing factors and the velocity of the flyer can better reveal the mechanism of detonation transmission. The influencing factors of lead azide are the diameter, height, density, and constraint conditions of the charge. In the MEMS detonation system, the diameter of the primary explosive is at the level of 1 mm [2,3,4,5,6,7,8,9], so the simulation range of the diameter should be about 1 mm; in [17,18], the output performance of lead azide with a charge height of 0.6 mm~3 mm was verified, so the charge height in the simulation was also set in this range. The common density of lead azide is more than 3 g·cm^−3^, and the crystal density is slightly larger than 4 g·cm^−3^, so the density range in the simulation was set to 2 g·cm^−3^~4 g·cm^−3^. At present, the commonly used metal and non-metal materials of the tube shell are aluminum, stainless steel, and organic glass; the materials (nickel, silicon, and copper) of S&A devices may also be used as the constraint materials of the primary explosive. The influence factors of the flyer were the thickness and material: the thickness of a polyimide flyer in an explosive foil initiator (EFI) is generally less than 100 μm, and the thickness of a flyer matched with a microcharge is generally larger than that of the EFI. So, the larger the charge, the greater the thickness of the matching flyer. In the simulation, the thickness of the flyer was designed to be 30 μm~200 μm. Polyimide is a commonly used material for EFIs; aluminum, copper, and stainless steel are also used as flyer materials for conventional charges, and titanium flyers are also used in some small detonators. Therefore, the performance of these types of flyers driven by a microcharge was evaluated. The main influencing factors of the acceleration chamber were the material and aperture: in an EFI, there is no S&A device, and the acceleration chamber is a separate device; its main materials are sapphire and stainless steel with high strength. In the MEMS detonation system, the flyer is sheared by the cover plate of the S&A device and accelerates in the detonating hole of the S&A device. At this time, the material used for the acceleration chamber is the same as that of the S&A (nickel, silicon, and copper); therefore, the performance of these materials as acceleration chambers was studied. The aperture of the acceleration chamber limited the diameter of the flyer, and there was an optimal matching relationship between the aperture of the acceleration chamber and the diameter of the lead azide. Considering that the common diameter of the lead azide was 0.9 mm, the aperture of the acceleration chamber was set to 0.3 mm~1.5 mm. Structural design parameters were optimized in combination with the shock initiation criterion.

#### 2.3.2. Simulation Model

**Figure 3:**
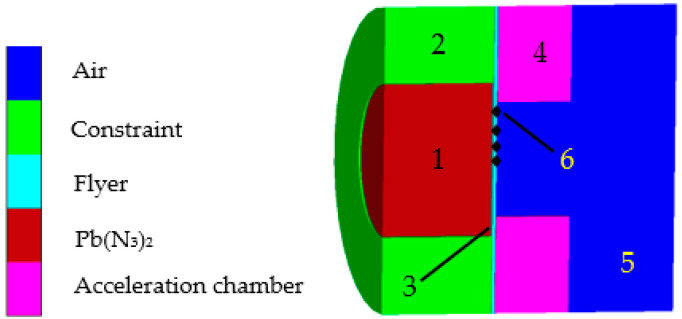
Simulation model of the microflyer driven by microsized lead azide: 1. Pb(N_3_)_2_; 2. constraint of the charge; 3. flyer; 4. acceleration chamber; 5. air; 6. Gauss point.

The simulation model of the microflyer driven by a microsized charge was established as shown in Figure 3. The Eulerian algorithm was applied to the explosive and air; the Lagrange algorithm was applied to the constraint of the lead azide, acceleration chamber, and flyer; the outflow boundary was applied to the air domain; the mesh size was 0.025 mm × 0.025 mm; and the Gauss points were distributed from the center of the flyer to the radial edge. The initiation point was located at the center of one side of the explosive.

The state equation and constitutive model were mainly used to describe the mechanical behavior of materials in the mechanical simulation of the explosion. The state equation described the thermodynamic behavior of the material, and the constitutive model considered the strength effect of the material. The Jones–Wilkins–Lee (JWL) state equation was used to describe the work behavior of the detonation products of the lead azide, and the acquisition of its parameters was based on the experimental data of the relationship between the density and detonation velocity of the lead azide [31]. The shock state equation and the Johnson–Cook constitutive model were used for the constraint, acceleration chamber, and titanium flyer, and the ideal gas state equation was used for air.

### 2.4. PDV Test

**Figure 4:**
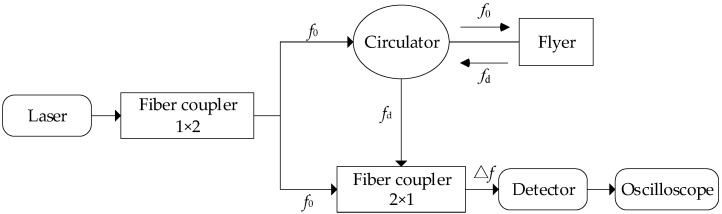
Schematic diagram of PDV.

In order to carry out simulation research, the accuracy of the simulation model should be evaluated by comparing the consistency of the flyer velocity–time curve in the simulation and test. The velocity of flyer is the most important parameter used to evaluate the initiation ability of the detonation sequence. Difficulties with the velocity test include the high transient process and the miniaturization of the target to be tested [32]. The PDV system uses the Doppler effect to calculate the velocity of moving objects according to the difference in frequency between the reflected laser frequency and the reference laser frequency. Compared with other speed-measurement methods, it has the advantages of a high precision, a wide range, and simple composition. The principle of the PDV system is shown in Figure 4.

The principle of the PDV system was as follows: the laser emitted by the laser source was divided into two channels by the fiber coupler, one of which was used as the reference laser that was shined into the next fiber coupler; the other entered the circulator and then irradiated the flyer, and the frequency changed after being reflected by the flyer, which was called the signal laser. The signal laser was transmitted to the next fiber coupler after passing through the circulator, and a frequency difference occurred with the reference laser that was detected by the detector. Finally, the frequency-difference signal was recorded by the oscilloscope.

The frequency of the reference laser and signal laser met the following relationship:

*f*_d_ = *f*_0_(1 + 2*v*_f_(*t*)/*C*) (14)

where *f*_0_ is the frequency of the reference laser in Hz, *f*_d_ is the frequency of the signal laser in Hz, *C* is the speed of light in km·s^−1^, and *v*_f_(*t*) is the velocity of the flyer changing with time in km·s^−1^. The frequency difference can be expressed as:

Δ*f* = *f*_d_ − *f*_0_ (15)

The light speed, frequency, and wavelength of the reference laser satisfied the following relationship:

*C* =*λ*_0_ × *f*_0_ (16)

where *λ*_0_ is the wavelength of the reference laser in nm.

When substituting Formulas (15) and (16) into Formula (14), the relationship between the flyer velocity *v*_f_(*t*) and frequency difference Δ*f* could be obtained:

(17) $$v_{f}(t)=\frac{f_{0}}{2C}(f_{d}-f_{0})=\frac{\lambda_{0}}{2}\Delta f$$

Therefore, the flyer velocity could be calculated by detecting the frequency difference of the laser caused by the flyer’s motion. Using a Fourier transform, the velocity–time curve of the flyer could be obtained by processing the frequency-difference signal Δ*f* with the MATLAB program.

**Figure 5:**
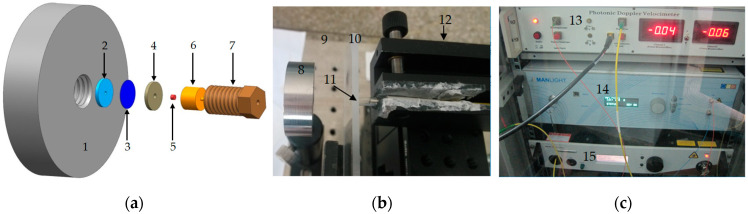
The PDV system of the microflyer driven by microsized lead azide: (**a**) test device of the microflyer driven by the microcharge; (**b**) position relationship between the test device and the optical fiber probe; (**c**) equipment of PDV system. 1. Fixture; 2. acceleration chamber; 3. titanium flyer; 4. constraint; 5. lead azide; 6. ignition device; 7. plug; 8. test device; 9. damping table; 10. organic glass sheet; 11. optical fiber probe; 12. the fixture of optical fiber probe; 13. PDV; 14. laser amplifier; 15. laser source.

The test system was mainly composed of the test device of the flyer driven by a microcharge, an organic glass sheet, an optical fiber probe, the PDV, a laser amplifier, and a laser source (Figure 5).

In Section 2.3.1, it was introduced that the diameter of the lead azide was at the 1 mm level, so the diameter of the lead azide in this experiment was submillimeter (0.9 mm); when the pressing pressure was 188 MPa, the corresponding theoretical density of 3.83 g·cm^−3^ was used as the charge density of the lead azide [19], which was also close to that used in the literature [17,18]. According to the preliminary simulation results, the flyer had a relatively high speed when the charge height was 1.2 mm, the thickness of the titanium flyer was 0.1 mm, and the aperture of the stainless steel acceleration chamber was 0.6 mm. Therefore, the flyer velocity–time curve under the above design parameters was obtained by the PDV test.

The test process was as follows:

1. Fix the optical fiber probe with a steel protective sleeve with the optical fiber probe clamp.
2. Fix the organic glass sheet on the path between the flyer and the probe to prevent the flyer from damaging the probe, and collect the flyer.
3. Assemble the flyer-type microdetonation sequence in the fixture, and fix the fixture position so that the optical fiber probe is aligned with the central axis of the acceleration chamber.
4. Check the continuity of the test circuit and oscilloscope settings, and fire after the inspection.
5. Read the original frequency difference signal from the oscilloscope, and obtain the flyer velocity–time curve through fast Fourier transform.

### 2.5. Steel Dent Test

According to the relationship between the design parameters and the velocity and detonation ability of the flyer, a prototype of the microdetonation sequence was designed. The main difference from the device used in the PDV test was that the SCB was placed on a printed circuit board (replacing the electrode plug), which reduced the volume, and then HNS-IV was added as a booster explosive.

After the test, the steel dent depth produced by the reaction of HNS-IV was used to verify whether the flyer had a reliable detonation function under the design parameters. When the prototype was placed on the steel block and the preset initiation energy was input, the following phenomena could occur in HNS-IV: a. the booster explosive was only knocked out of the pit by the flyer, and no steel dent is generated; b. deflagration of the booster explosive occurred, and some of the booster explosive was black, some of the booster explosive was burned out, and no steel dent is generated; c. most of the booster explosive was consumed, thereby resulting in a shallow steel dent; e. the booster explosive was completely consumed, thereby resulting in a deep steel dent, and the depth of the steel dent was consistent with the corresponding charge amount. Obviously, only the last case could show that the flyer had a reliable detonation capability and could verify the rationality of the previous parameter design.

**Figure 6:**
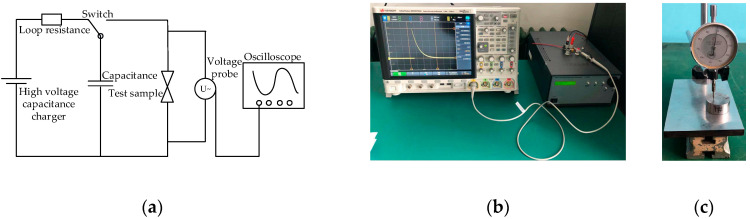
The equipment and connections in the schematic diagram of the steel dent test: (**a**) schematic diagram of test connections; (**b**) oscilloscope and charger; (**c**) steel dent tester.

The main equipment used in the steel dent test were: the microdetonation sequence, a high-voltage capacitance charger, a KEYSIGHT DSOX4104A oscilloscope, a VICTOR VC9807+ multimeter, a steel dent tester, and a steel block (φ 35 mm × 16 mm). The test principle and equipment are shown in Figure 6.

The main process of the steel dent test was as follows:

1. Set the firing voltage and oscilloscope parameters, then short-circuit the firing circuit.
2. Assemble the microdetonation sequence prototype.
3. Connect the prototype pin wire and firing circuit, remove the short-circuit cap of the pin wire, and close the explosion-proof box and test room.
4. Outside the test room, measure whether the resistance of the semiconductor bridge is within the normal range through the firing circuit.
5. Determine the test parameter settings again and ignite.
6. Save the ignition voltage data, record the status of samples, and measure the depth of the steel dent.

## 3. Results

### 3.1. Criterion of Flyer Impact Initiation

**Figure 7:**
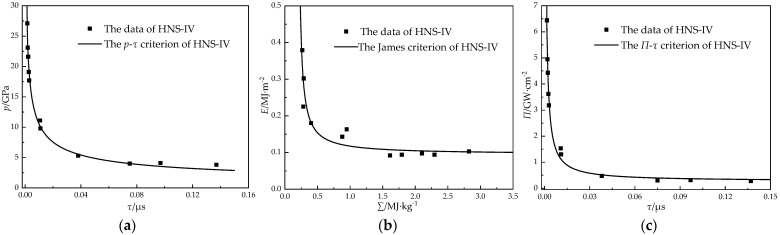
Three initiation criteria of HNS-IV: (**a**) *p*-*τ*; (**b**) James; (**c**) *Π*-*τ*.

The fitting results of the three initiation criteria of the HNS-IV are shown in Figure 7. As shown in Figure 7a, when the flyer impacted the explosive, the corresponding (*p*, *τ*) coordinate was located above the curve and then the explosive could be detonated; the farther away from the curve, the greater the corresponding initiation probability. Conversely, the explosive may not have been detonated, and the farther the data deviated from the curve, the lower the initiation probability. The overall fitting accuracy of the James criterion was lower than that of the other two criteria. When the particle-specific kinetic energy *Σ* increased, the curve approached the constant *E*_c_; when the particle specific kinetic energy *Σ* was low, the curve was close to the fixed value *Σ*_c_. The curves of the *Π*-*τ* criterion and the *p*-*τ* criterion were similar. When the impact time was longer, the energy power tended to be a customized *Π*_c_.

The *p*-*τ* initiation criterion of HNS-IV exhibited the following curve relationship:

*p*^2.0865^*τ* = 1.3584, *R*^2^ = 0.9884 (18)

The James initiation criterion of HNS-IV exhibited the following curve relationship:

(19) $$\frac{0.1916}{\Sigma }+\frac{0.095}{E}=1,\;R^{2}=0.8935$$

The *Π*-*τ* initiation criterion of HNS-IV exhibited the following curve relationship:

*Π* = 0.2715(1 + 0.0341/*τ*), *R*^2^ = 0.9879 (20)

The *p*-*τ* initiation criterion, the most classic criterion, is suitable for the one-dimensional ideal shock initiation model. The James and *Π*-*τ* initiation criteria introduced the particle specific kinetic energy *Σ*, power flux *Π*, and other parameters. Compared with the *p*-*τ* criterion, the type of booster explosive, the shape of flyer, and the pressure range were wider, which are the more commonly used initiation criteria. The *Π*-*τ* criterion is similar in form to the James initiation criterion, and according to the fitting results of the HNS-IV test data, the *Π*-*τ* initiation criterion had a higher fitting accuracy than the James criterion. Therefore, in the following, the *Π*-*τ* initiation criterion combined with the influencing factors of the microflyer driven by microsized lead azide will be used to analyze and assist in the design of the microdetonation sequence.

### 3.2. PDV Test

The velocity–time curves were obtained when the size of the lead azide was Φ 0.9 mm × 1.2 mm, the thickness of the titanium flyer was 0.1 mm, and the size of the stainless steel acceleration chamber was Φ 0.6 mm × 0.6 mm. The velocity–displacement curves of the flyer obtained via the simulation and the experiment were compared to verify the accuracy of the simulation model. 

**Figure 8:**
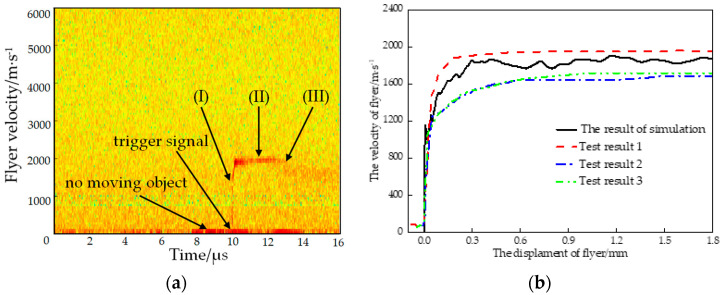
Comparison of the velocity of the flyer between the simulation and PDV test: (**a**) test results of the velocity–time curves of flyer; (**b**) comparison of simulation and test results.

Figure 8a shows three typical action processes of the microflyer driven by a microsized explosive: (I) the acceleration process of the flyer—the velocity of the flyer reached the maximum after about 0.5 μs of acceleration; (II) the stable flight process of the flyer—the flyer’s velocity remained stable within 2 μs~3 μs; (III) the collection process of the flyer—the flyer impacted on the organic glass sheet and was collected. Figure 8b shows the comparison of the simulation and test results for the flyer’s velocity–displacement curve. Table 3 shows the statistics of the flyer’s velocity and the root-mean-square error (RMSE).

In Figure 8b and Table 3, it can be seen that the velocity–displacement curves of the simulation and the test were in good agreement with the overall trend. There was a relative error between the test values. The reason for the divergence of the test results was that the distance between the optical fiber probe and the titanium flyer was great (about 40 mm in this test), so the laser path was more affected by external factors such as particles in the air in the process of incidence and reflection. The width of the laser beam was increased by the addition of organic glass, the error of the PDV system itself, and the dispersion of microcharge [33]. The ratio of the RMSE to the simulation value was 7.82%, which indicated that the simulation model had a certain accuracy and could be used to study the microflyer driven by a microsized charge.

### 3.3. Simulation of the Microflyer Driven by Microsized Lead Azide

After the simulation model of the microflyer driven by microsized lead azide was established and its accuracy was verified experimentally, a variety of design parameters were changed to calculate the velocity of flyer and other parameters. The relationship between the design parameters and the velocity of flyer was analyzed, and the optimum range of design parameters was obtained based on the *Π*-*τ* initiation criterion.

#### 3.3.1. Height of the Primary Explosive

**Figure 9:**
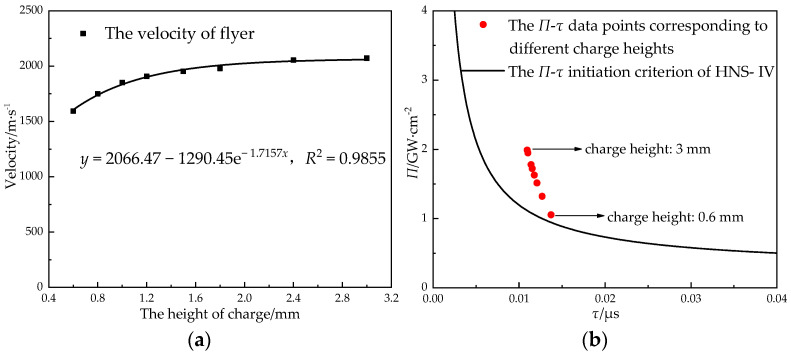
The relationship between the height of the charge, the velocity of the flyer, and the initiation criterion: (**a**) height of the charge and velocity of the flyer; (**b**) height of the charge and the initiation criterion.

The height of the primary explosive was an important factor that affected the velocity of the flyer and the axial height of the detonation sequence. In order to obtain the relationship between the charge height of the primary explosive and the velocity of the flyer, when the density of the lead azide was 3.83 g·cm^−3^ (without other instructions, the simulation density value of lead azide in this paper), the charge diameter was 0.9 mm, and the charge height was increased from 0.6 mm to 3 mm, the velocity of the Φ 0.6 mm × 0.1 mm titanium flyer was obtained by using a simulation, and the power flux *Π* and action time *τ* were calculated. Then, the analysis was carried out combined with the *Π*-*τ* initiation criterion of the HNS-IV. The results are shown in Figure 9.

When the diameter of the charge was constant, the output pressure was affected by the height of charge. With the increase in the height of charge, the output pressure increased rapidly at first. When the height of the charge reached a certain value, the growth rate of the output pressure decreased significantly, and the output pressure tended to be constant [17]. This rule was consistent with the simulation of the relationship between the height of the charge and the velocity of the flyer. The flyer’s velocity and power flux increased with the increase in the charge height, but the growth trend decreased gradually. In the simulation range, the flyer velocity tended to 2000 m/s.

From the relationship between the initiation criterion and the height of charge, it can be seen that the critical initiation condition of HNS-IV is satisfied when the height of charge is 0.6 mm, and the initiation probability increases with the increase in the height of charge. Considering the sensitivity of the booster explosive and the design requirement in reference [34] that the minimum output energy should be at least 25% higher than the minimum input energy required by the detonation sequence or the terminal device, the charge height of the booster explosive should not be less than 0.8 mm.

#### 3.3.2. Diameter of the Primary Explosive

**Figure 10:**
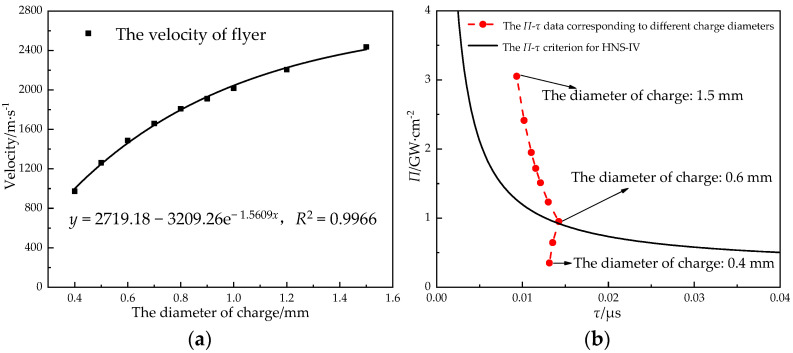
The relationship (**a**) between the velocity of the flyer and the diameter of the charge and (**b**) between the diameter of the charge and the initiation criterion.

For an explosive with a finite diameter, the detonation reaction is affected by the energy dissipation caused by lateral expansion. When the diameter of a charge is lower than the critical diameter, the detonation reaction cannot be transmitted. At the same time, when considering the safety and volume, the diameter of the charge should not be too large. Therefore, in the design of a microdetonation sequence, the diameter of the charge should be designed to be higher than its critical diameter and as small as possible to meet the detonation transmission capacity. The velocity and power flux of the Φ 0.6 mm × 0.1 mm titanium flyer were simulated and calculated when the charge height of the lead azide was 1.2 mm and the diameter of the charge was increased from 0.4 mm to 1.5 mm (Figure 10).

In the simulation range, the velocity of the flyer increased exponentially with the increase in the diameter of the lead azide. A diameter of 1.5 mm did not reach the limit diameter of the lead azide. The detonation parameters of the explosives increased continuously with the increase in the diameter of the charge, and the ability to drive the flyer was significantly enhanced, but the growth slope had a slowing trend. The influence of the charge diameter on the velocity of the flyer was greater than that of the charge height. The charge diameter of the lead azide was 0.6 mm for the critical initiation criterion of the HNS-IV. When considering the sensitivity, reliability, and volume, the diameter of the charge should not be less than 0.7 mm. A simulation study found in the literature [35] showed that when the charge height of copper azide was 0.5 mm, the charge diameter of the critical initiation of HNS-IV was between 0.6 mm and 0.7 mm.

#### 3.3.3. Density of the Primary Explosive

**Figure 11:**
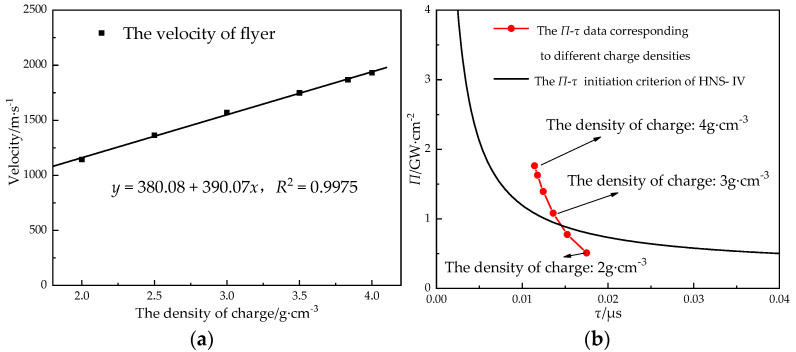
The relationship (**a**) between the velocity of the flyer and the density of the charge and (**b**) between the density of the charge and the initiation criterion.

The charge density is related to a variety of the detonation parameters of explosives; in numerical simulation, it also affects the parameter values of the JWL state equation. In order to study the relationship between the charge density and the velocity of the flyer, the power flux and the velocity of the flyer were calculated when the size of the lead azide was Φ 0.9 mm × 1.2 mm, the thickness of the titanium flyer was 100 μm, the aperture of the stainless steel acceleration chamber was 0.6 mm, and the charge density was 2 g·cm^−3^~4 g·cm^−3^. The results are shown in Figure 11. 

It can be seen in the simulation results that the charge density had a significant impact on the flyer’s velocity, which increased linearly with the increase in the density of the flyer. The higher the density of the charge, the greater the initiation probability; when the charge density of the lead azide was greater than 3 g·cm^−3^, it met the condition of initiating the HNS-IV.

The detonation velocity of high-density lead azide can exceed 5000 m/s [36]. However, in the process of shearing and driving the flyer, the energy of the lead azide is consumed. Moreover, the detonation product is affected by the rarefaction wave in the air domain, and its velocity decreases with the increase of the propagation distance and gradually approaches the velocity of flyer.

#### 3.3.4. Restraining Material of the Primary Explosive

The expansion of explosive detonation products is affected by lateral and axial sparse waves. When the shock wave impedance of the constraint material of the explosive is high, the influence of the lateral sparse wave is weakened, which helps to transfer the detonation energy.

In order to study the influence of the constraint material of lead azide on the velocity of the flyer, the constraint materials of the primary explosive were designed as follows: organic glass, silicon, aluminum, steel, copper, and nickel. The velocity of the flyer was simulated and calculated when the size of the lead azide was Φ 0.9 mm × 1.2 mm, the thickness of the titanium flyer was 0.1 mm, and the size of the stainless steel acceleration chamber was 0.6 mm × 1 mm.

**Figure 12:**
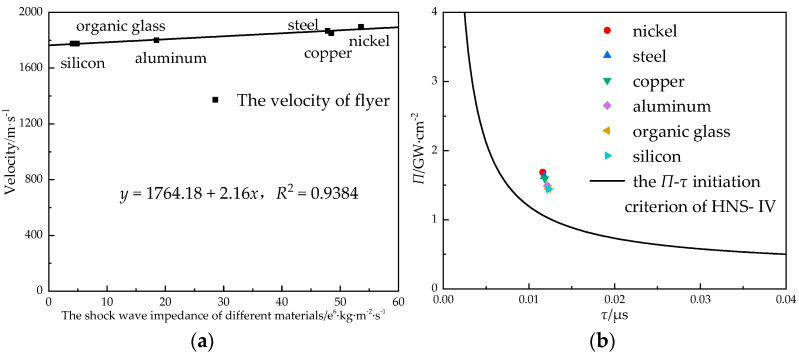
The relationship (**a**) between the velocity of the flyer and the restraint materials of the charge and (**b**) between the restraint materials of the charge and the initiation criterion.

The shock wave impedance that corresponded to different materials was calculated, and the relationship between the shock wave impedance and the velocity of flyer drawn as shown in Figure 12a. With the increase in the shock wave impedance, the velocity of the flyer increased linearly, but the overall increase was limited. The difference in the velocity of the flyer between the nickel material and the silicon material was 122 m/s. The larger the shock wave impedance, the higher the initiation probability; therefore, the material with a larger shock wave impedance should be selected as the constraint of initiating the explosive.

#### 3.3.5. Material of the Flyer

**Figure 13:**
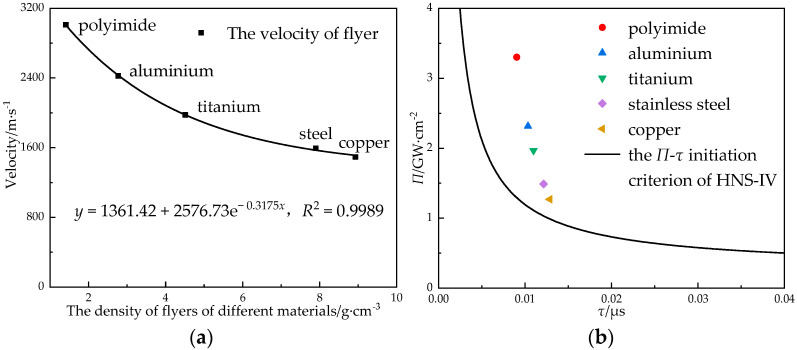
The relationship (**a**) between the velocity of the flyer and the density of the flyer and (**b**) between the materials of the flyer and the initiation criterion.

The density of the flyer material, the shear effect, the Hugoniot coefficient, and other characteristics affected the impact initiation effect of the booster explosive. In order to obtain the relationship between the flyer material and the velocity of flyer, the velocity of the flyer and the power flux of different materials with the lead azide (φ 0.9 mm × 1.8 mm in size) and the flyer (φ 0.6 mm × 0.1 mm in size) were simulated and calculated. The results are shown in Figure 13.

The density order of the flyers was polyimide < aluminum < titanium < steel < copper; the velocity of the flyer decreased exponentially with the increase in the density of the flyer. In the relationship between the power flux and the initiation criterion, it can be seen that the initiation probability of polyimide was the largest followed by the aluminum and titanium flyers. The initiation probabilities of the steel and copper flyers were only slightly higher than the critical initiation criterion; if the charge amount decreased, the initiation may have been unreliable.

**Figure 14:**
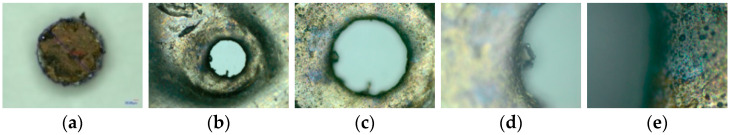
The morphology of the titanium flyer and the titanium sheet at different magnifications after the test: (**a**) 100× magnification; (**b**) 50× magnification; (**c**) 100× magnification; (**d**) 200× magnification; (**e**) 500× magnification.

**Figure 15:**
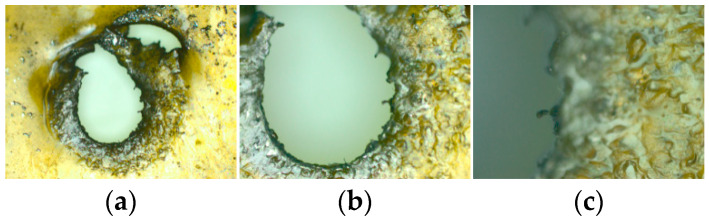
The morphology of the polyimide sheet at different magnifications after the test: (**a**) 50× magnification; (**b**) 100× magnification; (**c**) 500× magnification.

The titanium flyer and titanium sheet materials collected after the test are shown in Figure 14. The shape of the titanium flyer was basically unchanged, the mass loss was less, the shear was smoother, and the ablation phenomenon was slight. The shear effect of the polyimide under the action of the microcharge was not good (see Figure 15), the shape of the polyimide flyer was irregular, and the edge position was seriously ablated. The melting point of aluminum is only about 660 °C, while that of the other three metals is higher than 1000 °C. The shear-forming of the aluminum flyer was affected by the high temperature of the detonation products coupled with friction during flight, and it was difficult to ensure the complete shape of the aluminum flyer. In the actual shearing process, the copper flyers were not easily sheared due to their high density. Therefore, when considering the power flux and shear effect of the flyer, the titanium metal was the most suitable flyer material.

#### 3.3.6. Thickness of the Flyer

**Figure 16:**
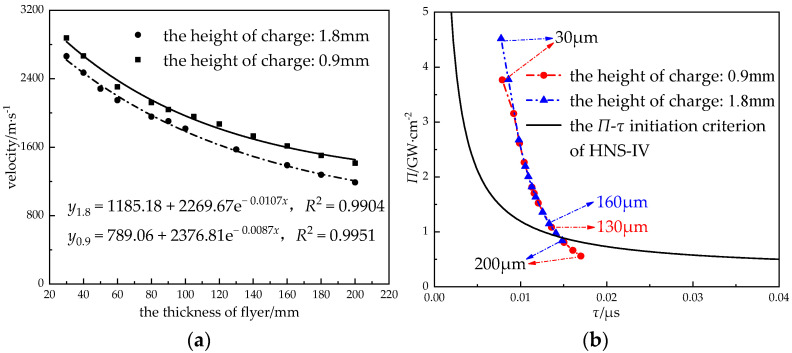
The relationship (**a**) between the thickness of flyer and the velocity of flyer and (**b**) between the thickness of the flyer and the initiation criterion.

The velocity of the flyer was related to the loading amount per unit area of charge on it; when the flyer was thin, the mass was light, the velocity of the flyer was large, and the shock wave pressure generated by the flyer’s impact on the explosive was large. The larger the thickness of the flyer, the longer the duration of the shock wave’s loading on the explosive, but the time was also related to the velocity, shape, and diameter of the flyer, as well as the Hugoniot parameters of the flyer and the explosive. In order to obtain the relationship between the thickness and the velocity of the flyer, the velocities of the titanium flyer with a diameter of 0.6 mm and a thickness of 0.03 mm~0.2 mm were simulated when the sizes of lead azide were Φ 0.9 mm × 0.9 mm and Φ 0.9 mm × 1.8 mm, respectively (see Figure 16).

The flyer velocity decreased exponentially with the increase in the flyer’s thickness. The power flux of the flyer increased with the decrease in the flyer’s thickness, and the initiation probability increased. When the thickness of the flyer was 30 μm~60 μm, the distance between the (*Π*, *τ*) data point of the flyer and the *Π*-*τ* criterion curve was close, and the initiation probability was close. When the height of charge was 0.9 mm, the thickness of the flyer that satisfied the initiation of the HNS-IV was 130 μm; when the charge height was 1.8 mm, the thickness of the flyer that satisfied the initiation of the HNS-IV is 160 μm. In practice, when the flyer was too thin and the charge was relatively large, the flyer was easy to break. Therefore, under the premise of satisfying the actual shear-forming effect, the thinner flyer should be selected.

#### 3.3.7. Material of the Acceleration Chamber

**Figure 17:**
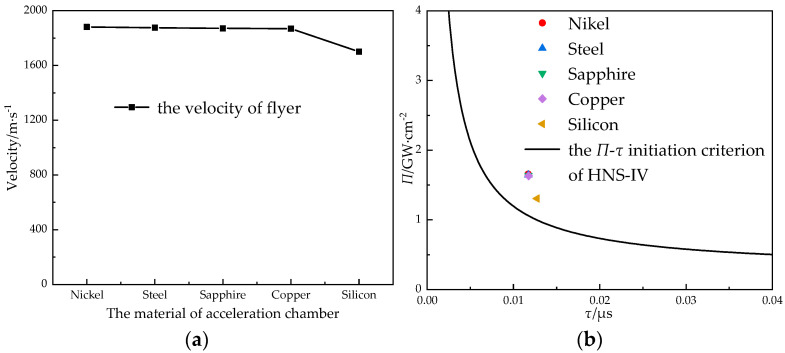
The relationship (**a**) between the material of the acceleration chamber and the velocity of the flyer and (**b**) between the material of the acceleration chamber and the initiation criterion.

As shown in Figure 17, the hole of the cover plate of the S&A device and the hole of the structural layer of the S&A device together constituted the acceleration chamber. The cover plate retained the gap for the movement of the structural layer, and a material with high hardness was selected for the cover plate of the S&A device, which was convenient for the flyer’s shear-forming. The cover plate and the structural layer could also be the same material. Nickel, copper, and silicon were the main materials of the S&A device; sapphire, manganese, and stainless steel are also widely used in acceleration chambers. In this section, the impact of the multilayer structure was not considered—only the impact of the material of the acceleration chamber on the velocity of the flyer. The materials of the acceleration chamber could be silicon, steel, nickel, copper, and sapphire. The velocity of flyer was calculated when the size of the lead azide was Φ 0.9 mm × 1.2 mm, the density of charge was 3.83 g·cm^−3^, the thickness of the titanium flyer was 100 μm, and the size of the acceleration chamber was Φ 0.6 mm × 1 mm (see Figure 17).

In the simulation results, it can be seen that the velocity and power flux of the flyer that corresponded to the metal and sapphire acceleration chambers were not significantly different, and the difference was small compared with the *Π*-*τ* initiation criterion of the HNS-IV. Compared with the other materials, the corresponding parameters of the silicon-based acceleration chamber decreased to a certain extent. The higher the hardness and smoothness of the acceleration chamber, the more conducive it was to the shear-forming and acceleration of the flyer. The order of hardness of the several materials was sapphire > nickel > silicon > stainless steel > copper. When considering the influence of the material hardness, copper should be excluded as the material of the acceleration chamber; and sapphire, nickel, and stainless steel should be selected as the materials of acceleration chamber.

#### 3.3.8. Aperture of the Acceleration Chamber

**Figure 18:**
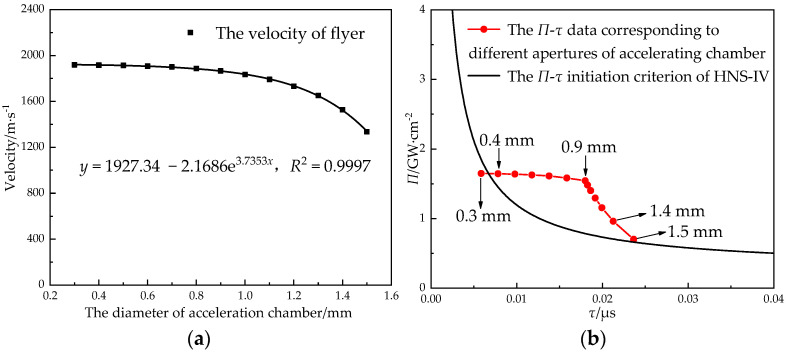
The relationship (**a**) between the aperture of the acceleration chamber and the velocity of the flyer and (**b**) between the aperture of the acceleration chamber and the initiation criterion.

The acceleration chamber and the detonation product of the lead azide caused the flyer to be shear-forming, while the size of the aperture of acceleration chamber limited the diameter of the flyer. In order to obtain the relationship between the aperture of the acceleration chamber and the flyer’s velocity, the velocity of the flyer was simulated when the aperture of the acceleration chamber was 0.3 mm~1.5 mm, the size of the lead azide was Φ 0.9 mm × 1.2 mm, and the thickness of the flyer is 0.1 mm (see Figure 18).

When the aperture of the acceleration chamber (0.3 mm~0.9 mm) was less than the diameter of the charge, the flyer’s velocity decreased slightly with the increase in the diameter of the acceleration chamber, and the flyer’s velocity was between 1866 m/s and 1918 m/s. When the aperture was 0.9 mm~1.1 mm, the velocity attenuation trend of the flyer began to increase. When the aperture (1.1 mm~1.5 mm) of the acceleration chamber was larger than the diameter of the charge, the velocity of the flyer decreased with the increase in the diameter of the acceleration chamber and the slope increased. When the aperture of acceleration chamber was larger than the diameter of the charge, in the initial shear stage, the flyer in the edge position was directly affected not by the detonation product but by the dragging effect of the flyer in the middle position to produce the speed. This energy consumption reduced the overall velocity of the flyer. With the increase in the aperture of the acceleration chamber, the quality of the flyer in the edge position increased, and the velocity of the flyer decreased obviously. At the same time, the aperture of the acceleration chamber could not be lower than the critical initiation diameter of the booster explosive.

With the increased aperture of the acceleration chamber, the initiation probability of the HNS-IV increased first and then decreased. When the aperture of the acceleration chamber and the diameter of the lead azide were the same, the (*Π*, *τ*) data points deviated the furthest from the *Π*-*τ* criterion curve, and the initiation probability was the largest. When the diameter of the acceleration chamber was 0.4 mm~1.4 mm, the initiation condition of the HNS-IV was satisfied. Therefore, the diameter of the acceleration chamber should be designed to be consistent with the aperture of the primary explosive.

### 3.4. Steel Dent Test

Based on previous research, a microdetonation sequence of a micro titanium flyer driven by microsized lead azide was designed within the optimal structural parameters. The ability of the microflyer to initiate the HNS-IV was verified by the steel dent test.

#### 3.4.1. Prototype of the Microdetonation Sequence

**Figure 19:**
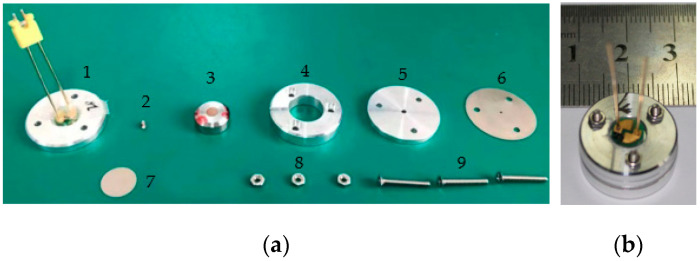
The microdetonation sequence: (**a**) component diagram; (**b**) prototype of microdetonation sequence. 1. ignition unit of semiconductor bridge; 2. lead azide; 3. HNS-IV; 4. base; 5. sleeve of lead azide; 6. acceleration chamber; 7. titanium sheet; 8. nuts; 9. bolts.

The microdetonation sequence is shown in Figure 19a. The ignition unit of the SCB was coated with LTNR. Due to the limited power provided by small-caliber ammunition, capacitor discharge was used. The ignition energy was 10 V/68 μF. 

According to the previous simulation results, the charge diameter had to be greater than 0.7 mm. Considering the literature [17] and the existing pressing mold, the charge diameter was designed to be 0.9 mm; the simulation and literature [17] showed that when the charge height was 1.8 mm, the output pressure of the lead azide and the corresponding flyer velocity tended to be stable; when considering that a certain margin had to be reserved and the volume of the prototype could not be too large, the charge height was designed as 1.8 mm. When the pressing pressure was designed as 188 Mpa, the corresponding theoretical charge density was 3.83 g·cm^−3^, which also met the detonation conditions. When considering the simulation results and the easy processing, stainless steel was used for the tube shell of the lead azide. The previous research results showed that titanium was the best flyer material and that the thickness of the flyer should be less than 160 μm. In order to ensure the integrity of the flyer after shearing, the thickness of the flyer could not be too small, so the thickness of the flyer was designed to be 80 μm (it possibly could be designed to be thinner). In the simulation, in addition to the silicon material, other materials had a similar effect when used for the acceleration chamber; in consideration of the easy processing, a stainless steel acceleration chamber was adopted. When the aperture of the acceleration chamber was 0.6 mm, the detonating condition was satisfied (the effect should be better when the design is consistent with the charge diameter).

The size of the HNS-IV was φ 3 mm × 3.7 mm, the density was 1.6 g·cm^−3^, the weight was about 42 mg, and the diameter of the sleeve was 8.9 mm. The HNS-IV charge was a small-volume model product, and the corresponding steel dent depth after complete detonation should have been greater than 0.3 mm. All components were connected with nuts and bolts. The assembled microdetonation sequence is shown in Figure 19b with an overall size of about φ 18.9 mm × 9 mm.

#### 3.4.2. Results of the Steel Dent Test

**Figure 20:**
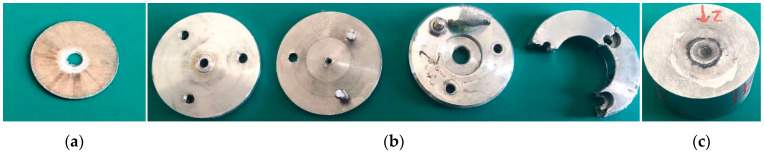
The sample after the test: (**a**) titanium sheet; (**b**) state of each component; (**c**) steel block for qualification.

The prototype after the test was as shown in Figure 20: the sheared part of the titanium sheet was relatively flat, the sleeve of the primary explosive was slightly expanded, the acceleration chamber was slightly deformed, the booster and the primary explosive were completely consumed, the base and bolts were cracked, and the steel qualification block had obvious dents.

The results of the steel dent test are shown in Table 4. The HNS-IV booster explosives were all detonated in the three tests. The average depth of the steel dent was 0.39 mm, and the average weight of the lead azide was 4.6 mg. The ability of the microflyer to initiate the HNS-IV was verified.

## 4. Discussion

The concept of the critical initiation criterion is similar to the concept of the energy absorbed by energetic materials [37], both of which involve the energy characteristics inside the explosives. It is worth noting that due to factors of the booster explosive and the test itself [37], it was better to leave a certain margin when using the critical initiation criterion for the design. Bowden and Guo obtained the *p*-*τ* and James criteria of HNS-IV by fitting [38,39], and Guo verified the accuracy of the criteria via simulation; the fitting accuracy of the James criterion was not high, and the *p*-*τ* criterion was proved to be more suitable for one-dimensional shock initiation. Tong obtained the *p*-*τ* criterion of HNS-IV by fitting the experimental [26] and theoretical data [40], and the applicable time obtained from the experimental data is within 0.0013 μs, which was less than 0.137 μs found in this study. Wang found the minimum all-firing voltage of HNS-IV initiated via EFI [41], obtained a flyer velocity that corresponded to the minimum all-firing voltage via PDV, and then obtained the all-firing *p*-*τ* initiation criterion of HNS-IV: the applicable maximum pressure was 13 GPa, which was less than the 27 GPa found in this study. The fitting range in this study was larger, and the fitting effects of the three common initiation criteria were compared. Compared with the *p*-*τ* criterion, the *Π*-*τ* criterion and the James criterion were more suitable for a wider range of explosive types, flyer shapes, pressures, and action times [23,24]. The fitting accuracy of the *Π*-*τ* criterion was higher than that of the James criterion, so it could be used to assist in the design of the microdetonation sequence.

In this paper, the relationship between the design parameters and initiation ability was revealed from the perspective of mechanics by combining the design parameters with an initiation criterion. In some of the literature, the influencing factors of flyers driven by different sizes and types of explosives were reported, but these influencing factors were not comprehensive enough and only the flyer velocity was obtained, so the detonation ability of the flyer could not be judged. Regarding the relationship between the velocity of the flyer driven by PBXN-5 and the density of the flyer (titanium, stainless steel, or copper), the diameter of the charge (1.5 mm~5 mm) and the thickness of the flyer (0.1 mm~0.5 mm) were studied via simulation [42], and the conclusions were consistent with those of this paper, which not only revealed the negative correlation between the flyer’s density and velocity, but also the morphology of the flyer after shearing. The sensitivity of PBNX-5 was relatively low, and the critical size was larger than that of the primary explosive, which was not suitable for driving the flyer as a primary charge. The test results showed that the BNCP with a size of φ 3 mm × 8 mm could drive a titanium flyer with a thickness of 0.1 mm to reach a speed of about 3600 m/s [13,43], but the charge size was quite different from that used in this study, and it was impossible to compare the difference between Pb(N_3_)_2_ and BNCP in driving the flyer. The simulation results showed that the velocity of the flyer with a diameter of 1 mm driven by Cu(N_3_)_2_ did not increase significantly after the charge height was 0.6 mm; in this study, the diameter of the Pb(N_3_)_2_ was 0.9 mm, and when the charge height was 1.8 mm, the velocity of the flyer tended to be stable, which was similar to the growth law of the output pressure of Pb(N_3_)_2_ [17]. Due to the high sensitivity of Cu(N_3_)_2_, it was difficult to test its output pressure. The experimental results confirmed that the titanium flyer could remain intact under the action of Cu(N_3_)_2_; while copper, aluminum, and polyimide showed different degrees of damage [14,15,16]. This study verified that under the action of Pb(N_3_)_2_, the titanium flyer could still remain intact while the polyimide flyer was incomplete; therefore, the matching of the titanium flyer and the microcharge was the best. Liu studied the relationship between the design parameters and the kinetic energy of a titanium flyer driven by PBXN-5 (diameter: 5 mm) [44], and Mu studied the relationship between the design parameters and the kinetic energy per unit area of a titanium flyer driven by lead azide (size: φ 1.2 mm × 2 mm) [45]. Compared with other studies, it was an improvement to use the kinetic energy of the flyer or the kinetic energy per unit area as a characterization parameter, but it was still rough and directly affected the relevant conclusions because the energy absorbed by the explosives only accounted for a portion of the kinetic energy of the flyer [37]. In the current study, it was more reasonable to use the incident shock wave parameters formed in the explosive as the initiation criterion when the flyer struck the explosive. In summary, the previous studies only focused on the velocity, kinetic energy, or kinetic energy per unit area of the flyer, and these three parameters could not accurately reflect the initiation ability of the flyer. In the current paper, the initiation mechanism could be revealed more clearly by combining the design parameters with an initiation criterion. Moreover, the design parameters used in the current paper were more comprehensive.

In this study, a prototype of HNS-IV detonated by a titanium flyer driven by microsized Pb(N_3_)_2_ was designed. The steel dent test confirmed that the prototype had a reliable detonation transmission function. The purpose of the design of the prototype was to make the size or charge amount of the primary explosive as small as possible under the premise of meeting the detonation transmission function. The U.S. military used Ag(N_3_)_2_ (φ 2 mm × 0.5 mm; 6 mg), Cu(N_3_)_2_, or energetic ink explosives to drive flyers to detonate EDF-11 explosives [3,4,5,6,7,8]. The Kaman Company used a slurry of Pb(N_3_)_2_ to drive a flyer to detonate HNS-IV explosives [9]. The volume and charge of Ag(N_3_)_2_ used by the U.S. military were larger than that of the Pb(N_3_)_2_ used in the current study (φ 0.9 mm × 1.8 mm and 4.6 mg, respectively), but the charge height was smaller. This may have been because the sensitivity of EDF-11 is higher than that of HNS-IV, and the in situ process maturity of the U.S. military is high. Kaman’s design idea was consistent with that of this paper, but the literature we found did not introduce specific design parameters. In the literature [12], when the size and weight of Cu(N_3_)_2_ were φ 2 mm × 1.5 mm and 7.5 mg, respectively, a titanium flyer with a thickness of 30 μm~50 μm could be driven to detonate a CL-20-based booster explosive. The size and charge amount of Cu(N_3_)_2_ used in the literature were larger than that of the Pb(N_3_)_2_ used in the current paper, which may have been related to the in situ synthesis process. Wang used lead azide (φ 1.6 mm × 2 mm; 14.4 mg) to drive a stainless steel flyer (with a thickness of 30 μm) to detonate HNS-IV [10]; in the latest research results published by their group, the size and charge of the lead azide (φ 1.2 mm × 2 mm; 8.1 mg) were further reduced [46], which was close to the level used in the current study. The size or charge amount of the Pb(N_3_)_2_ used in this paper can be further reduced based on the following aspects: (a) increase the charge diameter appropriately to reduce the charge height; (b) reduce the thickness of the flyer properly to improve the power flux of the flyer; (c) design the diameter of the acceleration chamber to be designed closer to the diameter of the charge; and (d) encapsulate the outside of the prototype to reduce the leakage of the detonation products.

## 5. Conclusions

According to the shock-initiation theory, the *Π*-*τ* initiation criterion of HNS-IV was fitted. The simulation model of the microflyer driven by microsized lead azide was established. The ratio of the RMSE to the simulation value was 7.82%.

Based on the *Π*-*τ* initiation criterion, the relationships between various design parameters and the velocity of the flyer, the power flux, and the action time were studied; the results were as follows. Titanium was the best flyer material; based on the premise of satisfying the forming effect, the thinner flyer should be selected; the aperture of accelerating chamber should be consistent with the diameter of the primary explosive; sapphire and metal were the most suitable materials for the acceleration chamber; the material with a high shock wave impedance should be selected for the restraint of the primary explosive; and the velocity and power flux of the flyer increased linearly or exponentially with the increase in the density, diameter, and height of the primary explosive.

A microdetonation sequence for HNS-IV detonated by a titanium microflyer driven by microsized lead azide was designed. When the weight of the lead azide was 4.6 mg, the microflyer successfully detonated the HNS-IV, and the average depth of the steel dent was 0.39 mm.

## Figures and Tables

**Table 1:** The critical initiation velocities of the HNS-IV flyers.

*δ*_f_/μm	Material of Flyer	*v*_f_/km·s^−1^	*δ*_f_/μm	Material of Flyer	*v*_f_/km·s^−1^
25 ^1^	Polyimide	2.96	3.0	Aluminum ^1^	3.66
25		2.84	3.5		3.30
76		1.84	4.0		3.16
140		1.51	4.5		2.92
165		1.53	5.0		2.77
254		1.46			

^1^ The data in the first line for the polyimide flyer and the data for the aluminum flyer came from [26]; the other experimental data for the polyimide flyer came from [27].

**Table 2:** The Hugoniot parameters of the HNS-IV and flyers.

Type	*ρ*/g·cm^−3^	*A*/km·s^−1^	*S*
HNS-IV [28]	1.60	1.430	2.630
Ti [29]	4.51	5.220	0.767
Al [28]	2.79	1.290	5.370
Kapton [30]	1.41	2.737	1.410

**Table 3:** Comparison between simulation and PDV test results for the velocity of flyer.

Test Number	Test Value /m·s^−1^	Simulation Value /m·s^−1^	RMSE /m·s^−1^	RMSE/Simulation Value /%
1	1960	1868	146	7.82
2	1687			
3	1716			

**Table 4:** Results of the steel dent test.

Test Number	Weight of the Lead Azide/mg	Depth of the Steel Dent/mm
1	4.7	0.411
2	4.5	0.359
3	4.7	0.399
Mean value	4.6	0.390

## Data Availability

Data available on request, having regard to restrictions, e.g., privacy or ethical. The data presented in this study are available on request from the corresponding author.

## Appendix A

**Table A1:** Symbols and explanations.

Symbol	Explanation	Unit
*p*	Shock wave pressure	GPa
*u*	Shock wave particle velocity	km·s^−1^
*τ*	Shock wave action time	μs
*D*	Shock wave velocity	km·s^−1^
*E*	Energy density	MJ·m^−2^
*Σ*	Specific kinetic energy	MJ·kg^−1^
*Π*	Power flux	GW·cm^−2^
*A*	Intercept of Hugoniot line	km·s^−1^
*S*	Slope of Hugoniot line	Dimensionless
*v*_f_	Flyer velocity	km·s^−1^
*d*_f_	Flyer diameter	μm
*δ*_f_	Flyer thickness	μm
*C*_e_	Sound velocity	km·s^−1^
*f*_0_	Frequency of reference laser	Hz
*f*_d_	Frequency of signal laser	Hz
*λ*_0_	Wavelength of the reference laser	nm
